# Supplementary material for: Survey on Psychosocial Conditions of Official Veterinarians in Germany: Comparison with Other Professions and Differences between Age Groups, Gender, and Workplace Characteristics
Source: Animals (Basel). 2024 Jul 3;14(13):1975. doi: 10.3390/ani14131975 (PMC11240587; doi:10.3390/ani14131975)
Supplement: Supplementary file 1 [file animals-14-01975-s001.zip › Supplementary material S2_questionnaire.pdf]

LimeSurvey

Sehr geehrte Kolleginnen und Kollegen,

Herzlichen Dank für Ihr Interesse an dieser Umfrage!

Die Umfrage hat das Ziel, die psychosoziale Belastung von Amtstierärzt:innen/ amtlichen Tierärzt:innen zu erheben. Gleichzeitig sollen lösungsorientiert auch die Verfügbarkeit von Maßnahmen zur Steigerung des Wohlbefindens und Ihre Einschätzung zum Nutzen dieser Maßnahmen erfasst werden.

Es dauert etwa 20 Minuten den Fragebogen auszufüllen. Bitte nehmen Sie sich die Zeit und füllen Sie ihn bis zum Ende aus. Die Ergebnisse sollen einen etwaigen Handlungsbedarf aufzeigen und können dazu beitragen, dass in Zukunft sich auch Ihr Arbeitsplatz positiv verändert.

Die Fragen beziehen sich zumeist auf Ihr persönliches Erleben – bitte füllen Sie sie subjektiv bezogen auf Ihre aktuelle Arbeitssituation als amtliche Tierärztin bzw. amtlicher Tierarzt aus. Bitte beachten Sie, dass der Fragebogen auch eventuell belastende Fragen, etwa zu Gewalterfahrungen, beinhaltet.

Herzlichen Dank für Ihre Teilnahme!

Charlotte Jensen

Dr. med. vet. K. Charlotte Jensen

Institut für Veterinär-Epidemiologie und Biometrie

FB Veterinärmedizin, Freie Universität Berlin

Charlotte.Jensen@fu-berlin.de

unterstützt durch den

BbT - Bundesverband der beamteten Tierärzte e.V.

Teil A: Angaben zu Ihrer Person und Ihrem Arbeitsplatz

A1. Sind Sie derzeit als amtlicher Tierarzt/ amtliche Tierärztin beschäftigt?

bei Krankschreibung/ Elternzeit/ Referendariat/ Teilzeit etc. bitte ebenfalls Ja anklicken

Ja ☐

Nein ☐

A2. In welchem Bereich sind Sie überwiegend tätig?

Tierschutz ☐

Tierseuchen ☐

Schlachttier- und Fleischuntersuchungen (SFU) ☐

Lebensmittelsicherheit (außer SFU) ☐

Tierarzneimittel ☐

Tierische Nebenprodukte ☐

Versuchstierschutz ☐

Sonstiges ☐

A3. Welchem Geschlecht fühlen Sie sich zugehörig?

weiblich ☐

männlich ☐

divers ☐

A4. Wie alt sind Sie?

34 Jahre oder jünger ☐

35-44 Jahre ☐

45-54 Jahre ☐

55 Jahre oder älter ☐

A5. Wieviele Jahre sind Sie bereits als amtliche Tierärztin/ amtlicher Tierarzt tätig?

LimeSurvey

A6. Welche Weiterbildungen haben Sie absolviert?

- ein etwa zweijähriges Referendariat ☐
- Fachtierarzt Öffentliches Veterinärwesen ☐
- Fachtierarzt Lebensmittel ☐
- Master Veterinary Public Health ☐
- eine mehrmonatige Weiterbildung für den öffentlichen Veterinärdienst ☐
- Sonstiges ☐
- derzeit in Weiterbildung ☐
- keine spezifische Weiterbildung ☐

A7. Bitte geben Sie an, welche sonstigen Weiterbildungen Sie absolviert haben.

A8. Bitte geben Sie an, für welche Weiterbildung(en) Sie sich derzeit in Ausbildung befinden.

A9. In welchem Bundesland sind Sie (überwiegend) tätig?

- Baden Württemberg ☐
- Bayern ☐
- Berlin ☐
- Brandenburg ☐
- Bremen ☐
- Hamburg ☐
- Hessen ☐
- Mecklenburg-Vorpommern ☐
- Niedersachsen ☐
- Nordrhein Westfalen ☐
- Rheinland-Pfalz ☐
- Saarland ☐
- Sachsen ☐
- Sachsen-Anhalt ☐
- Schleswig-Holstein ☐
- Thüringen ☐
- Sonstiges ☐

Sonstiges

A10. Auf welcher Ebene sind Sie tätig?

- Untere Veterinärbehörde (Landkreis/ Bezirksamt) ☐
- Mittlere Veterinärbehörde (bspw. LAVES) ☐
- Ministerium ☐

A11. Haben Sie Führungsaufgaben für andere amtliche Tierärzt:innen (Führungskraft)?

- Ja ☐
- Nein ☐

LimeSurvey

A12. **Welches Anstellungsverhältnis trifft auf Sie zu?**

- verbeamtet ☐
- unbefristet angestellt ☐
- befristet angestellt ☐
- Sonstiges ☐

Sonstiges

[ ]

A13. **Wieviele Wochenstunden arbeiten Sie wöchentlich als amtliche Tierärztin/ Tierarzt laut Arbeitsvertrag?**

[| | | | | | | | |]

A14. **Arbeiten Sie mindestens einmal...**

	ja	nein
... pro Monat am Wochenende oder einem Feiertag?	☐	☐
... pro Woche abends (nach18:30 Uhr) oder nachts (vor 5:00 Uhr)?	☐	☐
... pro Woche von zuhause aus?	☐	☐
... pro Woche von unterwegs aus/ führen Kontrollen vor Ort aus?	☐	☐

Teil B: Anforderungen bei der Arbeit

B1. **Die folgenden Fragen betreffen die Anforderungen bei der Arbeit.**

	immer	oft	manchmal	selten	nie/ fast nie
Müssen Sie sehr schnell arbeiten?	☐	☐	☐	☐	☐
Arbeiten Sie den ganzen Tag im hohen Tempo?	☐	☐	☐	☐	☐
Kommt es vor, dass Sie nicht genügend Zeit haben, alle Ihre Aufgaben zu erledigen?	☐	☐	☐	☐	☐
Kommen Sie mit Ihrer Arbeit in den Rückstand?	☐	☐	☐	☐	☐
Müssen Sie Überstunden machen?	☐	☐	☐	☐	☐
Gehört es zu Ihrer Arbeit, sich mit den persönlichen Problemen anderer Menschen zu beschäftigen?	☐	☐	☐	☐	☐
Müssen Sie bei der Arbeit viele Dinge gleichzeitig im Blick behalten?	☐	☐	☐	☐	☐

LimeSurvey

	immer	oft	manchmal	selten	nie/ fast nie
Erfordert Ihre Tätigkeit, dass Sie schwierige Entscheidungen treffen?	☐	☐	☐	☐	☐
Erfordert Ihre Tätigkeit, dass Sie viele Informationen aus Ihrer Erinnerung abrufen müssen?	☐	☐	☐	☐	☐
Bringt Ihre Tätigkeit Sie in emotional verstörende Situationen?	☐	☐	☐	☐	☐

Teil C: Emotionale Anforderungen und Work-Life-Balance

C1. Inwieweit stimmen Sie den folgenden Aussagen zu?

	in sehr hohem Maße	in hohem Maße	zum Teil	in geringem Maße	in sehr geringem Maße
Meine Arbeit ist emotional fordernd.	☐	☐	☐	☐	☐
Meine Arbeit verlangt, dass ich meine Gefühle verberge.	☐	☐	☐	☐	☐
Meine Arbeit beansprucht so viel Energie, dass sich dies negativ auf mein Privatleben auswirkt.	☐	☐	☐	☐	☐
Meine Arbeit beansprucht so viel Zeit, dass sich dies negativ auf mein Privatleben auswirkt.	☐	☐	☐	☐	☐

Teil D: Einflussmöglichkeiten und Spielraum bei der Arbeit

D1. Die folgenden Fragen beziehen sich auf Ihre Einflussmöglichkeiten und Ihren Spielraum bei der Arbeit.

	immer	oft	manchmal	selten	nie/ fast nie
Haben Sie großen Einfluss auf Entscheidungen, die Ihre Arbeit betreffen?	☐	☐	☐	☐	☐
Haben Sie Einfluss auf die Menge der Arbeit, die Ihnen übertragen wird?	☐	☐	☐	☐	☐
Haben Sie Einfluss darauf, was Sie bei Ihrer Arbeit tun?	☐	☐	☐	☐	☐
Können Sie selbst bestimmen, wann Sie eine Pause machen?	☐	☐	☐	☐	☐
Haben Sie einen Einfluss darauf, WIE Sie Ihre Arbeit machen?	☐	☐	☐	☐	☐
Können Sie Ihren Urlaub mehr oder weniger dann nehmen, wenn Sie möchten?	☐	☐	☐	☐	☐
Können Sie Ihren Arbeitsplatz ohne besondere Erlaubnis für eine halbe Stunde verlassen, wenn private Angelegenheiten dies erfordern?	☐	☐	☐	☐	☐

Teil E: Entwicklungsmöglichkeiten und Bedeutsamkeit

E1.

	in sehr hohem Maße	in hohem Maße	zum Teil	in geringem Maße	in sehr geringem Maße
Haben Sie die Möglichkeit durch Ihre Arbeit neue Dinge zu lernen?	☐	☐	☐	☐	☐
Können Sie Ihre Fertigkeiten oder Ihr Fachwissen bei Ihrer Arbeit anwenden?	☐	☐	☐	☐	☐
Finden Sie Ihre Arbeit sinnvoll?	☐	☐	☐	☐	☐
Würden Sie anderen Tierärzt:innen empfehlen, sich bei Ihrer Behörde zu bewerben?	☐	☐	☐	☐	☐
Haben Sie das Gefühl, dass Ihre Arbeitsstelle für Sie von großer Bedeutung ist?	☐	☐	☐	☐	☐
Genießen Sie es, anderen von Ihrer Arbeit zu erzählen?	☐	☐	☐	☐	☐

Teil F: Regelungen und Ablauf bei der Arbeit

F1. Nun einige Fragen zu Regelungen und Abläufen bei Ihrer Arbeit.

	in sehr hohem Maße	in hohem Maße	zum Teil	in geringem Maße	in sehr geringem Maße
Werden Sie rechtzeitig im Voraus über Veränderungen an Ihrem Arbeitsplatz informiert, z.B. über wichtige Entscheidungen, Veränderungen oder Pläne für die Zukunft?	☐	☐	☐	☐	☐
Erhalten Sie alle Informationen, die Sie brauchen, um Ihre Arbeit gut zu erledigen?	☐	☐	☐	☐	☐
Gibt es klare Ziele für Ihre Arbeit?	☐	☐	☐	☐	☐
Wissen Sie genau, was von Ihnen bei der Arbeit erwartet wird?	☐	☐	☐	☐	☐
Werden bei Ihrer Arbeit widersprüchliche Anforderungen gestellt?	☐	☐	☐	☐	☐
Müssen Sie manchmal Dinge tun, die eigentlich auf andere Weise getan werden sollten?	☐	☐	☐	☐	☐
Müssen Sie manchmal Dinge tun, die Ihnen unnötig erscheinen?	☐	☐	☐	☐	☐

Teil G: Kolleg:innen und Führung

G1. Bitte schätzen Sie ein, in welchem Maße Ihre unmittelbare Führungskraft...

	in sehr hohem Maße	in hohem Maße	zum Teil	in geringem Maße	in sehr geringem Maße	ich habe keine Führungskraf t
... der Arbeitszufriedenheit einen hohen Stellenwert beimisst?	☐	☐	☐	☐	☐	☐

	in sehr hohem Maße	in hohem Maße	zum Teil	in geringem Maße	in sehr geringem Maße	ich habe keine Führungskraft
... die Arbeit gut plant?	☐	☐	☐	☐	☐	☐
... Konflikte gut löst?	☐	☐	☐	☐	☐	☐

G2. Die folgenden Fragen betreffen Ihr Verhältnis zu Ihren Kolleg:innen und zu Ihrer Führungskraft.

	immer	oft	manchmal	selten	nie/ fast nie	keine Führungskraft/ Kolleg:innen
Wie oft erhalten Sie bei Bedarf Hilfe und Unterstützung von Ihren Kolleg:innen?	☐	☐	☐	☐	☐	☐
Wie oft erhalten Sie im Bedarfsfall Hilfe und Unterstützung von Ihrer unmittelbaren Führungskraft?	☐	☐	☐	☐	☐	☐
Ist die Atmosphäre zwischen Ihnen und Ihren Arbeitskolleg:innen gut?	☐	☐	☐	☐	☐	☐

G3. Die nächsten Fragen beziehen sich nicht auf Ihre eigene Tätigkeit, sondern auf den Arbeitsplatz an sich.

	in sehr hohem Maße	in hohem Maße	zum Teil	in geringem Maße	in sehr geringem Maße
Vertraut das Management/ die Führung darauf, dass die Mitarbeiter:innen ihre Arbeit gut machen?	☐	☐	☐	☐	☐
Können die Mitarbeitenden den Informationen vertrauen, die vom Management/ der Führung kommen?	☐	☐	☐	☐	☐
Werden Konflikte auf gerechte Weise gelöst?	☐	☐	☐	☐	☐
Wird die Arbeit gerecht verteilt?	☐	☐	☐	☐	☐
Erfährt Ihre Arbeit Anerkennung und Wertschätzung durch das Management/ die Führung?	☐	☐	☐	☐	☐
Werden die Vorschläge der Mitarbeitenden von der Führung ernst genommen?	☐	☐	☐	☐	☐

Teil H: Sicherheit des Arbeitsplatzes

H1. Machen Sie sich Sorgen, dass...

	in sehr hohem Maße	in hohem Maße	zum Teil	in geringem Maße	in sehr geringem Maße
... Sie arbeitslos werden?	☐	☐	☐	☐	☐
... es schwierig für Sie wäre, eine neue Arbeit zu finden, wenn Sie arbeitslos würden?	☐	☐	☐	☐	☐
... man Sie gegen Ihren Willen auf eine andere Position versetzen könnte?	☐	☐	☐	☐	☐

Teil I: Zufriedenheit

I1. Wenn Sie Ihre jetzige Arbeitssituation betrachten, wie zufrieden sind Sie mit...

	sehr zufrieden	zufrieden	teils-teils	unzufrieden	sehr unzufrieden
... Ihrer Arbeit insgesamt unter Berücksichtigung aller Umstände?	☐	☐	☐	☐	☐
... Ihrem Lohn/ Gehalt?	☐	☐	☐	☐	☐
... Ihren Berufsperspektiven?	☐	☐	☐	☐	☐
... der Art und Weise, wie Ihre Abteilung geführt wird?	☐	☐	☐	☐	☐

Teil J: Gesundheit

J1. Die kommenden Fragen betreffen Ihren Gesundheitszustand und Ihr Wohlbefinden. Bitte versuchen Sie nicht zwischen Symptomen zu unterscheiden, die arbeitsbedingt sind, und solchen, die andere Gründe haben. Bitte beschreiben Sie Ihren generellen Zustand in den letzten vier Wochen.

Wenn Sie den besten denkbaren Gesundheitszustand mit 10 Punkten bewerten und den schlechtesten denkbaren mit 0 Punkten: Wie viele Punkte vergeben Sie dann für Ihren derzeitigen Gesundheitszustand? Bitte geben Sie die entsprechende Zahl an.

- 0 ☐
- 1 ☐
- 2 ☐
- 3 ☐
- 4 ☐
- 5 ☐
- 6 ☐
- 7 ☐
- 8 ☐
- 9 ☐
- 10 ☐

LimeSurvey

J2. **Energie und psychisches Wohlbefinden:**

Wenn Sie an die letzten vier Wochen denken: wie häufig...

	immer	oft	manchmal	selten	nie/ fast nie
.. waren Sie körperlich erschöpft?	☐	☐	☐	☐	☐
... waren Sie emotional erschöpft?	☐	☐	☐	☐	☐
... waren Sie von Ihrer Arbeit begeistert?	☐	☐	☐	☐	☐
... haben Sie schlecht oder unruhig geschlafen?	☐	☐	☐	☐	☐
... hatten Sie Probleme, sich zu entspannen?	☐	☐	☐	☐	☐
... hatten Sie Bauchschmerzen?	☐	☐	☐	☐	☐
... hatten Sie Kopfschmerzen?	☐	☐	☐	☐	☐
... hatten Sie Probleme sich zu konzentrieren?	☐	☐	☐	☐	☐
... waren Sie traurig?	☐	☐	☐	☐	☐
... haben Sie das Interesse an alltäglichen Dingen verloren?	☐	☐	☐	☐	☐
... haben Sie sich ausgebrannt gefühlt?	☐	☐	☐	☐	☐
... waren Sie angespannt?	☐	☐	☐	☐	☐
... hatten Sie Schwierigkeiten, Entscheidungen zu treffen?	☐	☐	☐	☐	☐
... hatten Sie ein schlechtes Gewissen oder haben sich schuldig gefühlt?	☐	☐	☐	☐	☐
... fehlte Ihnen Selbstvertrauen?	☐	☐	☐	☐	☐

J3. **Selbst-Wirksamkeit: Wie gut treffen diese Aussagen auf Sie als Person zu?**

	trifft voll und ganz zu	trifft eher zu	trifft etwas zu	trifft nicht zu
Wenn die Menschen gegen mich sind, finde ich Wege, das zu erreichen, was ich möchte.	☐	☐	☐	☐
Ich bin zuversichtlich, dass ich unerwartete Ereignisse regeln kann.	☐	☐	☐	☐

Teil K: Konflikte und Gewalt

K1. **Welche Situationen empfinden Sie bei Ihrer Tätigkeit als besonders belastend?**

K2. **Waren Sie im Rahmen Ihrer Tätigkeit in den letzten 12 Monaten Tratsch oder übler Nachrede ausgesetzt?**

Ja, täglich ☐
Ja, wöchentlich ☐
Ja, monatlich ☐
Ja, mehrere Male ☐
Nein ☐

K3. **Wenn ja, durch wen?**

Kolleg:innen ☐
Führungskräfte ☐
Tierhalter:innen ☐
Lebensmittelhersteller:innen ☐
Sonstiges ☐

Sonstiges

K4. **Waren Sie in Rahmen Ihrer Tätigkeit in den letzten 12 Monaten in Streit oder Konflikte involviert?**

Ja, täglich ☐
Ja, wöchentlich ☐
Ja, monatlich ☐
Ja, mehrere Male ☐
Nein ☐

LimeSurvey

K5. **Wenn ja, durch wen?**

- [ ] Kolleg:innen
- [ ] Führungskräfte
- [ ] Tierhalter:innen
- [ ] Lebensmittelhersteller:innen
- [ ] Sonstiges

Sonstiges

K6. **Wurden Sie in den letzten 12 Monaten aus einem arbeitsbezogenen Anlass in sozialen Medien (z.B. Facebook), per Email oder Textnachrichten belästigt?**

- [ ] Ja, täglich
- [ ] Ja, wöchentlich
- [ ] Ja, monatlich
- [ ] Ja, mehrere Male
- [ ] Nein

K7. **Wenn ja, durch wen?**

- [ ] Kolleg:innen
- [ ] Führungskräfte
- [ ] Tierhalter:innen
- [ ] Lebensmittelhersteller:innen
- [ ] Sonstiges

Sonstiges

K8. **Wurden Sie in den letzten 12 Monaten in Rahmen Ihrer Tätigkeit sexuell belästigt?**

- Ja, täglich ☐
- Ja, wöchentlich ☐
- Ja, monatlich ☐
- Ja, mehrere Male ☐
- Nein ☐

K9. **Wenn ja, durch wen?**

- Kolleg:innen ☐
- Führungskräfte ☐
- Tierhalter:innen ☐
- Lebensmittelhersteller:innen ☐
- Sonstiges ☐

Sonstiges

K10. **Wurden Ihnen in den letzten 12 Monaten in Rahmen Ihrer Tätigkeit Gewalt angedroht?**

- Ja, täglich ☐
- Ja, wöchentlich ☐
- Ja, monatlich ☐
- Ja, mehrere Male ☐
- Nein ☐

K11. **Wenn ja, durch wen?**

- Kolleg:innen ☐
- Führungskräfte ☐
- Tierhalter:innen ☐
- Lebensmittelhersteller:innen ☐

Sonstiges

Sonstiges

K12. Wurden Ihnen in den letzten 12 Monaten in Rahmen Ihrer Tätigkeit körperliche Gewalt zugefügt?

- Ja, täglich
- Ja, wöchentlich
- Ja, monatlich
- Ja, mehrere Male
- Nein

K13. Wenn ja, durch wen?

- Kolleg:innen
- Führungskräfte
- Tierhalter:innen
- Tiere
- Lebensmittelhersteller:innen
- Sonstiges

Sonstiges

Teil L: Maßnahmen zum Wohlbefinden

L1. Sofern Sie Vor-Ort-Kontrollen durchführen: Welche der folgenden Maßnahmen stehen Ihnen in der Regel bzw. regelmäßig zur Verfügung und welche halten Sie für sinnvoll?

	steht zur Verfügung	steht nicht zur Verfügung
Kontrollen nur mit Begleitperson (4-Augen-Prinzip)		
Deeskalationstraining		

LimeSurvey

	steht zur Verfügung	steht nicht zur Verfügung
Unterstützung bei schwierigen Situationen durch Polizei/ Ordnungsamt	☐	☐
Selbstverteidigungskurs	☐	☐
Stichsichere Westen	☐	☐

L2. Sofern Sie Vor-Ort-Kontrollen durchführen: Welche der folgenden Maßnahmen stehen Ihnen in der Regel bzw. regelmäßig zur Verfügung und welche halten Sie für sinnvoll?

	sinnvoll	nicht sinnvoll
Kontrollen nur mit Begleitperson (4-Augen-Prinzip)	☐	☐
Deeskalationstraining	☐	☐
Unterstützung bei schwierigen Situationen durch Polizei/ Ordnungsamt	☐	☐
Selbstverteidigungskurs	☐	☐
Stichsichere Westen	☐	☐

L3. Welche weiteren Maßnahmen halten Sie zum Eigenschutz bei Kontrollen für sinnvoll?

L4. Welche Maßnahmen für das psychische Wohlbefinden werden Ihnen durch Ihren Arbeitgeber zur Verfügung gestellt bzw. halten Sie für sinnvoll?

	wird angeboten	wird nicht angeboten
regelmäßige Teammeetings	☐	☐
kollegiale Fallberatung	☐	☐
Supervision (durch externe Personen)	☐	☐
regelmäßiger Austausch mit anderen Behörden (sozialpsychiatrischer Dienst, Jugendamt o.ä.)	☐	☐
Kurse für Entspannungstechniken (z.B. autogenes Training, Meditation, Achtsamkeit)	☐	☐
Sportkurse (z.B. Volleyball, Rückentraining)	☐	☐
Kurse im Bereich Konfliktmanagement und Kommunikation	☐	☐
Resilienztraining	☐	☐

	wird angeboten	wird nicht angeboten
Psychotherapeutische Angebote	☐	☐

L5. Welche Maßnahmen für das psychische Wohlbefinden werden Ihnen durch Ihren Arbeitgeber zur Verfügung gestellt bzw. halten Sie für sinnvoll?

	sinnvoll	nicht sinnvoll
regelmäßige Teammeetings	☐	☐
kollegiale Fallberatung	☐	☐
Supervision (durch externe Personen)	☐	☐
regelmäßiger Austausch mit anderen Behörden (sozialpsychiatrischer Dienst, Jugendamt o.ä.)	☐	☐
Kurse für Entspannungstechniken (z.B. autogenes Training, Meditation, Achtsamkeit)	☐	☐
Sportkurse (z.B. Volleyball, Rückentraining)	☐	☐
Kurse im Bereich Konfliktmanagement und Kommunikation	☐	☐
Resilienztraining	☐	☐
Psychotherapeutische Angebote	☐	☐

L6. Bitte sortieren Sie die folgenden Maßnahmen hinsichtlich Management und Führung danach, wie sie Ihrer Meinung nach geeignet sind, das Wohlbefinden an Ihrem Arbeitsplatz zu steigern. (Rang 1 = höchste Wichtigkeit)

mehr Stellen für tierärztliches Personal schaffen	☐
mehr Stellen für Verwaltungspersonal schaffen	☐
mehr Stellen für sonstiges Personal schaffen	☐
Arbeitsabläufe effizienter gestalten (bspw. hinsichtlich Aktenführung, Priorisierung der Aufgaben)	☐
Personalwechsel besser gestalten (Zeit für Einarbeitung, schnellere Bewerbungsverfahren)	☐
Aufgaben umverteilen (gleichmäßiger oder nach persönlichen Interessen)	☐
technische Ressourcen bereitstellen (bspw. Tablets für mobiles Arbeiten)	☐
Kommunikation und Arbeitsklima verbessern (bspw. Teammeetings, Mediation)	☐

L7. **Haben Sie weitere Ideen für Maßnahmen, um die psychosoziale Belastung zu reduzieren?**

L8. **Haben Sie bei Ihrer derzeitigen Arbeitsstelle bereits eine Überlastungsanzeige gestellt?**

Nein ☐

Ja, einmalig ☐

Ja, bereits mehr als einmal ☐

L9. **Wurden daraufhin Gegenmaßnahmen durch die Führung ergriffen?**

Nein ☐

Ja, und zwar die folgenden ☐

Teil M: Abschluss

M1. **Welche Anmerkungen oder Kommentare haben Sie noch zu Ihrer persönlichen Situation? Gibt es Aspekte, die in dieser Umfrage nicht erfasst wurden?**

M2. **Haben Sie noch Anmerkungen zum Fragebogen oder der Befragung allgemein?**

Herzlichen Dank für Ihre Teilnahme!

Bei Fragen oder Anregungen schreiben Sie mir gerne eine E-Mail: Charlotte.Jensen@fu-berlin.de

Sollten die Fragen Sie aufgewühlt oder belastet haben und Sie nehmen bei sich Anzeichen von Depression, Burnout oder anderen psychischen Erkrankungen wahr, vertrauen Sie sich den Menschen in Ihrer Umgebung an und nehmen Sie Hilfe in Anspruch!

Hier einige Ideen für Hilfsangebote: TelefonSeelsorge (0800 1110111), Hausarzt/ Hausärztin, https://vetivolution.org/, sozialpsychiatrischer Dienst, psychiatrische Kliniken/ psychiatrischer Notdienst, Selbsthilfegruppen
