# Supplementary material for: Survey on Psychosocial Conditions of Official Veterinarians in Germany: Comparison with Other Professions and Differences between Age Groups, Gender, and Workplace Characteristics
Source: Animals (Basel). 2024 Jul 3;14(13):1975. doi: 10.3390/ani14131975 (PMC11240587; doi:10.3390/ani14131975)
Supplement: Supplementary file 1 [file animals-14-01975-s001.zip › Supplementary material Table S1.pdf]

animals

Table S1: Questions of the COPSOQ III used in the survey assessing the psychosocial situation of official veterinarians in Germany. Bold captions indicate the dimension to which the items belong. Level indicates the level of the COPSOQ III (core, middle, long or additional). GSV indicates if the question is part of the German Standard Version (Yes; No).

Code	Level	Question	GSV?*
	**Quantitative Demands**		
QD2	core	How often do you not have time to complete all your work tasks?	Yes
QD3	core	Do you get behind with your work?	Yes
	**Work Pace**		
WP1	core	Do you have to work very fast?	Yes
WP2	core	Do you work at high pace throughout the day?	Yes
	**Cognitive Demands**		
CD1	long	Do you have to keep your eyes on lots of things while you work?	No
CD2	long	Does your work require that you remember a lot of things?	No
CD4	long	Does your work require you to make difficult decisions?	No
	**Emotional Demands**		
ED1	middle	Does your work put you in emotionally disturbing situations?	No
EDX2	core	Do you have to deal with other people's personal problems as part of your work?	Yes
ED3	core	Is your work emotionally demanding?	Yes
	**Demands for Hiding Emotions**		
HE2	middle	Does your work require that you hide your feelings?	Yes
	**Influence at Work**		
INX1	core	Do you have a large degree of influence on the decisions concerning your work?	Yes
IN3	middle	Can you influence the amount of work assigned to you?	Yes
IN4	middle	Do you have any influence on what you do at work?	Yes
IN6	middle	Do you have any influence on HOW you do your work?	No
	**Possibilities for Development**		
PD2	core	Do you have the possibility of learning new things through your work?	Yes
PD3	core	Can you use your skills or expertise in your work?	Yes
	**Control over Working Time**		
CT1	middle	Can you decide when to take a break?	Yes
CT2	middle	Can you take holidays more or less when you wish?	Yes
CT4	middle	If you have some private business is it possible for you to leave your place of work for half an hour without special permission?	No
CT5	middle	Do you have to do overtime?	Yes
	**Meaning of Work**		
MW1	core	Is your work meaningful?	Yes
	**Predictability**		
PR1	core	At your place of work, are you informed well in advance concerning for example important decisions, changes or plans for the future?	Yes
PR2	core	Do you receive all the information you need in order to do your work well?	Yes
	**Recognition**		
RE1	core	Is your work recognized and appreciated by the management?	Yes
	**Role Clarity**		

CL1	core	Does your work have clear objectives?	Yes
CL3	middle	Do you know exactly what is expected of you at work?	Yes
	**Role Conflicts**		
CO2	core	Are contradictory demands placed on you at work?	Yes
CO3	core	Do you sometimes have to do things which ought to have been done in a different way?	Yes
	**Illegitimate Tasks**		
IT1	middle	Do you sometimes have to do things which seem to be unnecessary?	Yes
	**Quality of Leadership**		
QL2	long	To what extent would you say that your immediate superior gives high priority to job satisfaction?	Yes
QL3	core	To what extent would you say that your immediate superior is good at work planning?	Yes
QL4	core	To what extent would you say that your immediate superior is good at solving conflicts?	Yes
	**Social Support from Supervisor**		
SSX2	core	How often do you get help and support from your immediate superior, if needed?	Yes
	**Social Support from Colleagues**		
SCX1	core	How often do you get help and support from your colleagues, if needed?	Yes
	**Sense of Community at Work**		
SW1	core	Is there a good atmosphere between you and your colleagues?	Yes
	**Commitment to Workplace**		
CW1	long	Do you enjoy telling others about your place of work?	Yes
CW2	long	Do you feel that your place of work is of great importance to you?	Yes
CW3	long	Would you recommend other people to apply for a position at your workplace?	No
	**Work Engagement**		
WE1	long	I am enthusiastic about my job.	Yes
	**Job Insecurity**		
JI1	core	Are you worried about becoming unemployed?	Yes
JI3	core	Are you worried about it being difficult for you to find another job if you became unemployed?	Yes
	**Insecurity over Working Conditions**		
IW1	core	Are you worried about being transferred to another job against your will?	Yes
	**Job Satisfaction**		
JS1	middle	Regarding your work in general. How pleased are you with your work prospects?	Yes
JS4	core	Regarding your work in general. How pleased are you with your job as a whole, everything taken into consideration?	Yes
JS5	middle	Regarding your work in general. How pleased are you with your salary?	Yes
new	new	Regarding your work in general. How pleased are you with the way your department is leaded?	Yes
	**Work Life Conflict**		
WF2	core	Do you feel that your work drains so much of your energy that it has a negative effect on your private life?	Yes
WF3	core	Do you feel that your work takes so much of your time that it has a negative effect on your private life?	Yes

	**Vertical Trust**		
TM1	core	Does the management trust the employees to do their work well?	Yes
TMX2	core	Can the employees trust the information that comes from the management?	Yes
	**Organizational Justice**		
JU1	core	Are conflicts resolved in a fair way?	Yes
JU3	long	Are all suggestions from employees treated seriously by the management?	No
JU4	core	Is the work distributed fairly?	Yes
	**Gossip and Slander**		
GS1	long	Have you been exposed to gossip and slander at your workplace during the last 12 months?	No
GS2	long	If yes, by whom?	No
	**Conflicts and Quarrels**		
CQ1	long	Have you been involved in quarrels or conflicts at your workplace during the last 12 months?	No
New	long	If yes, by whom?	No
	**Cyber Bullying**		
HSM1	long	Have you been exposed to work-related harassment on the social media (e.g. Facebook), by e-mail or text messages during the last 12 months?	No
HSM2	long	If yes, by whom?	No
	**Sexual Harassment**		
SH1	long	Have you been exposed to undesired sexual attention at your workplace during the last 12 months?	No
SH2	long	If yes, by whom?	No
	**Threats of Violence**		
TV1	long	Have you been exposed to threats of violence at your workplace during the last 12 months?	No
TV2	long	If yes, by whom?	No
	**Physical Violence**		
PV1	long	Have you been exposed to physical violence at your workplace during the last 12 months?	No
PV2	long	If yes, by whom?	No
	**Self Rated Health**		
GH2	long	If you evaluate the best conceivable state of health at 10 points and the worst at 0 points: how many points do you then give your present state of health?	Yes
	**Sleeping Troubles**		
SL1	long	How often have you slept badly and restlessly?	
	**Burnout**		
BO1	long	How often have you felt worn out?	Yes
BO2	long	How often have you been physically exhausted?	Yes
BO3	long	How often have you been emotionally exhausted?	Yes
	**Stress**		
ST1	long	How often have you had problems relaxing?	No
ST3	long	How often have you been tense?	No
	**Somatic Stress**		
SO1	long	How often have you had stomach ache?	No
SO2	long	How often have you had a headache?	No

	**Cognitive Stress**		
CS1	long	How often have you had problems concentrating?	No
CS3	long	How often have you had difficulty in taking decisions?	No
	**Depressive Symptoms**		
DS1	long	How often have you felt sad?	No
DS2	long	How often have you lacked self-confidence?	No
DS3	long	How often have you had a bad conscience or felt guilty?	No
DS4	long	How often have you lacked interest in everyday things?	No
	**Self-Efficacy**		
SE2	long	If people work against me, I find a way of achieving what I want.	No
SE4	long	I feel confident that I can handle unexpected events.	No
